# Prognostic Significance of Circulating Tumor Cells in Non-Small-Cell Lung Cancer Patients: A Meta-Analysis

**DOI:** 10.1371/journal.pone.0078070

**Published:** 2013-11-04

**Authors:** Jianwei Huang, Ke Wang, Jianjun Xu, Jian Huang, Tao Zhang

**Affiliations:** 1 Department of Oncology, Second Affiliated Hospital, Zhejiang University School of Medicine, Hangzhou, China; 2 School of Finance, Zhejiang University of Finance and Economics, Hangzhou, China; 3 Department of Preventive and Health Care, Second Affiliated Hospital, Zhejiang University School of Medicine, Hangzhou, China; Institute of Experimental Endocrinology and Oncology 'G. Salvatore' (IEOS), Italy

## Abstract

**Background:**

The prognostic significance of circulating tumor cells (CTCs) detected in patients with non-small-cell lung cancer (NSCLC) is still inconsistent. We aimed to assess the prognostic relevance of CTCs using a meta-analysis.

**Methods:**

We searched PubMed, Web of Science and EMBASE for relevant studies that assessed the prognostic relevance of CTCs in NSCLC. Statistical analyses were conducted to calculate the summary incidence, odds ratio, relative risks (RRs) and 95% confidence intervals (CIs) using fixed or random-effects models according to the heterogeneity of included studies.

**Results:**

A total of 20 studies, comprising 1576 patients, met the inclusion criteria. In identified studies, CTCs were not correlated with histology (adenocarcinoma vs squamous cell carcinoma) (odds ratio [OR]  =  0.88; 95% confidence interval [CI]: 0.59–1.33; Z = –0.61; *P* = 0.545). However, pooled analyses showed that CTCs were associated with lymph node metastasis (OR = 2.06; 95% CI: 1.18–3.62; Z = 2.20; *P* = 0.027) and tumor stage (OR  = 1.95; 95% CI: 1.08–3.54; Z = 2.53; *P* = 0.011). Moreover, CTCs were significantly associated with shorter overall survival (relative risk [RR]  = 2.19; 95% CI: 1.53–3.12; Z = 4.32; *P*<0.0001) and progression-free/disease-free survival (RR  = 2.14; 95% CI: 1.36–3.38; Z = 3.28; *P*<0.0001).

**Conclusion:**

The presence of CTCs indicates a poor prognosis in patients with NSCLC. Further well-designed prospective studies are required to explore the clinical applications of CTCs in lung cancer.

## Introduction

Primary lung cancer is the leading cause of cancer-related death worldwide, and non-small-cell lung cancer (NSCLC) accounts for 85% of those deaths [1]. The frequent occurrence of distant metastasis is the main cause of high mortality. Although histology and stage are currently used the prognostic factor in NSCLC patient follow-up, 25% to 50% of patients with early-stage NSCLC show tumor recurrence [2]. Moreover, even in patients without clinically detectable distant metastasis at the time of initial diagnosis, distant micrometastasis may frequently develop during diagnosis, even undergoing surgery. Therefore, sensitive prognostic and predictive markers is urgently needed in lung clinical oncology. In NSCLC patients, detecting circulating tumor cells (CTCs) may show clinical benefits in diagnosis and treatment.

CTCs are tumor cells that are shed from the primary tumor, flowing through the bloodstream and circulate throughout the body. The first report on metastatic tumor cells in drainage veins was by Ashworth in 1869 [3]. In the recent years, various new CTC assays are developed and employed for their detection, including immunocytochemistry (ICC), reverse-transcriptase polymerase chain reaction (RT-PCR), and the CellSearch System [4]–[6]. Among various detect methods, the CellSearch system is introduced for clinical use by the U.S. FDA. This system uses immunomagnetic purification with antibodies against epithelial cell adhesion molecule (EpCAM) from peripheral blood. Regarding specificity of the CellSearch, it is the use of EpCAM expression as mechanism for detecting CTCs. Consequently, CTCs that low or absent expression EpCAM are easy missed. Several papers have demonstrated alternative detection technologies on the basis of EpCAM or other antigen with apparent greater sensitivity than CellSearch [7], [8], and further studies for larger clinic are warranted.

Recently, a pooled analysis in breast and colorectal cancer has demonstrated the prognostic significance of CTCs, and changes in CTC number with standard therapy has highlighted the potential of CTCs [9], [10]. However, in NSCLC patients, there still remains controversial regarding the incidence of CTCs and the clinical significance in NSCLC cancer. Some studies have reported that tumor cell detection in the blood is significantly associated with shorter survival [11], [12]. In contrast, other studies have failed to show such an association between the presence of CTCs and a poorer prognosis [13]. Therefore, to address the prognostic value of CTCs in patients with NSCLC, we conducted a meta-analysis to determine the association of CTCs status with clinicopathological parameters, including histology, tumor stage, lymph node metastasis, and patients’ survival.

## Materials and Methods

### Publication search

We conducted an independent review of citations from PubMed (http://www.ncbi.nlm.nih.gov/pubmed/) between 1 January 2000, and 1 January 2013. Key words were circulating tumor cell(s), lung cancer, or lung neoplasm. The search strategy used text terms such as circulating cancer cells, CTCs, blood epithelial cell, and non-small cell lung cancer to identify relevant information as well. We also carried out independent searches using Web of Science and EMBASE databases, to ensure that no articles were overlooked. Furthermore, relevant articles were identified from cited references of retrieved articles and review articles by a manual search.

### Eligibility criteria

Studies were included in the meta-analysis according to the following: (1) when the presence of CTC status and either disease stage or survival (overall survival [OS] and/or progression-free survival [PFS]) data of NSCLC were investigated; (2) when investigators provided relevant information for estimating odds ratios (ORs) or relative risks (RRs); and (3) when the same authors reported a series of results in multiple publications, only the latest was included in the analysis. Studies that did not report any clinical outcome were excluded. Studies with fewer than 20 analyzed patients, reviews, and comments were also excluded.

### Data extraction

We recorded the following information from each eligible paper: author’s name, patient’s country, publication year, number of patients, tumor stage, methods of CTC detection, detection rate, and cutoff value of CTCs. According to the study objective, we performed two types of analysis: the first analysis determined whether CTC status is correlated with clinicopathological parameters, which included histology (adenocarcinoma vs squamous cell carcinoma), lymph node metastasis, and cancer stage (T_1_+T_2_ vs T_3_+T_4_). The second analysis determined whether CTC status is associated with OS or PFS/disease-free survival (DFS).

### Statistical analysis

Survival data of each study were collected from original papers or calculated as described by Parmar et al [14]. The pooled ORs and RRs with 95% confidence intervals (CIs) for survival were calculated by fixed or random-effects models. Heterogeneity between studies was evaluated with the Cochran’s Q test and *P* values. When *P* was less than 0.05, a random-effects estimate was used. Otherwise, a fixed-effects model estimate was presented. Publication bias was assessed using the funnel plot and the Egger’s test, and the “trim-and-fill” method was to evaluate the effect of publication bias on the pooled effect. All statistical analyses were conducted using the software R/metafor version 2.14.0. *P* values less than 0.05 were considered statistically significant.

## Results

### Description of studies

**Figure 1:**
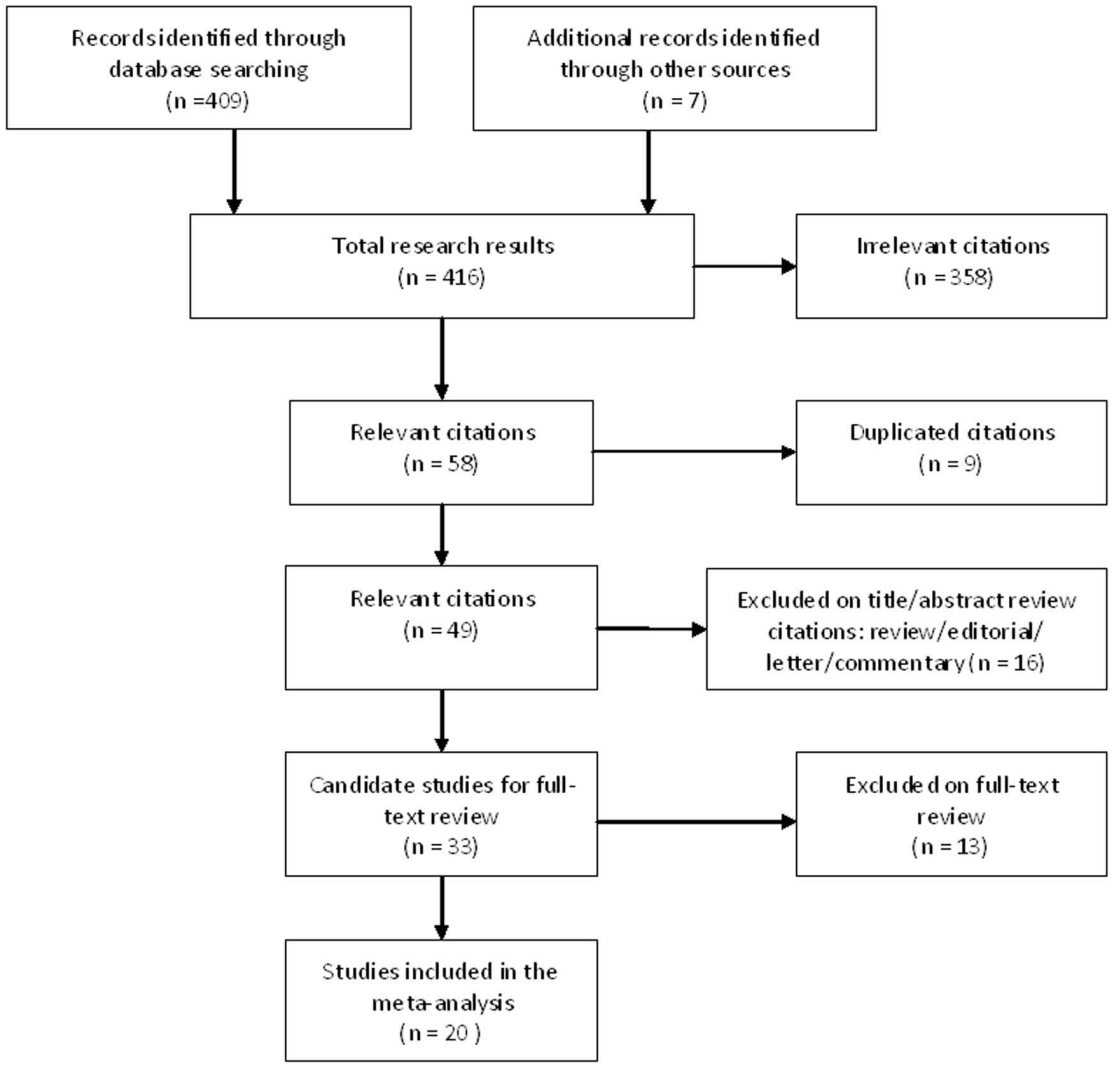
Flow chart of selecting the eligible publications.

The present work followed the guidelines for systematic reviews and meta-analyses (PRISMA) (Checklist S1). The systematic literature search yielded a total of 416 records (Figure 1). After screening of titles, abstracts, and full text version, 396 articles were excluded because of irrelevant publications, review articles, duplicates, and overlapped studies. Finally, 20 publications met the criteria for analysis, comprising 1576 patients [2], [11]–[13], [15]–[30]. The sample size per study ranged from 24 to 208 patients, and studies were published between 2002 and 2012. The main characteristics of the included studies are summarized in Table 1. There were three types of methods for assessment of CTCs status in blood specimens: RT-PCR, ICC, and the CellSearch system. 8 studies used RT-PCR techniques to evaluate CTCs status, and 8 studies used ICC techniques. These studies using epithelial cell antigen were varied, including CEA, CK, ERCC1 and other antigen. Four studies used CellSearch method, the cutoff of CTCs were set at ≥1 or ≥2 per 7.5 ml blood.

**Table 1:** Main characteristics of the eligible studies.

Study	Patient's country	Year	Tumor stage	Technique	Number of patients	Detection rate %	Target antigen /target gene	Sampling time	Cutoff of CTC+
Yamashita	Japan	2002	I–III	RT-PCR	103	62(60%)	CEA	Pre-TM or Post-TM	--
Sienel	Germany	2003	I–III	other ICC	62	11(18%)	CK	Pre-TM	1 CTC/2.5×10^6^ MNC
Huang	China	2004	I-IV	other ICC	58	20(34.5%)	--	Pre-TM, Intro-TM or Post-TM	--
Sher	China	2005	I-IV	RT-PCR	54	39(72%)	CK19, Trim28, Ubiquitin- thiolesterase	--	--
Rolle	Germany	2005	I–III	other ICC	29	25(85%)	Human- epithelial antigen	Pre-TM or Post-TM	--
Sheu	China	2006	I-IV	RT-PCR	100	90(90%)	Multimarker assay	Pre-TM	--
Chen	China	2007	I-IIIb	RT-PCR	62	44(66%)	CK19	Pre-TM or Post-TM	--
Liu	China	2008	I–IV	RT-PCR	134	84.3%	TSA-9, KRT-19, Pre-proGRP	--	--
Tanaka	Japan	2009	I–IV	CellSearch	125	(30.6%)	--	Pre-TM or Intro-TM	1 CTCs/7.5 ml
Yie	China	2009	I–IV	RT-PCR	143	63(44%)	Survivin	Pre-TM	--
Hofman	France	2010	I–IV	other ICC	208	102(49%)	--	Pre-TM	50 CNHCs
Funaki	Japan	2011	I–IV	other ICC	94	68(72%)	CK	Post-TM	1 CTC/1×10^6^
Krebs	United Kingdom	2011	III–IV	CellSearch	101	21(21%)	--	Pre-TM or Post-TM	2 CTCs/7.5 ml
Yoon	Korea	2011	I–III	RT-PCR	79	26(42.6%)	TTF-1,CK19	Pre-TM or Post-TM	--
Das	America	2012	IV	other ICC	57	24(42%)	ERCC1	Pre-TM or Post-TM	2 CTCs//1×10^6^
Punnoose	Australia	2012	IV	CellSearch	37	28(78%)	--	Pre-TM or Post-TM	1 CTC/7.5 ml
Isobe	Japan	2012	IV or Recurrence	CellSearch	24	8(33.3%)	--	--	1 CTCs/7.5 ml
Hirose	Japan	2012	IV	CellSearch	33	12(36.3%)	--	Pre-TM or Post-TM	1 CTCs/7.5 ml
Franco	Italy	2012	I–IV	other ICC	45	11(24.4%)	CK	Post-TM	--
Nieva	America	2012	I–IV	other ICC	28	45(68%)	CK	Pre-TM or Post-TM	1 CTCs/ml

*Abbreviations ICC  = * Immunocytochemical, RT-PCR  =  Reverse transcriptase polymerase chain reaction, TM  =  Treatment

### Correlation of CTCs with clinicopathological parameters

**Figure 2:**
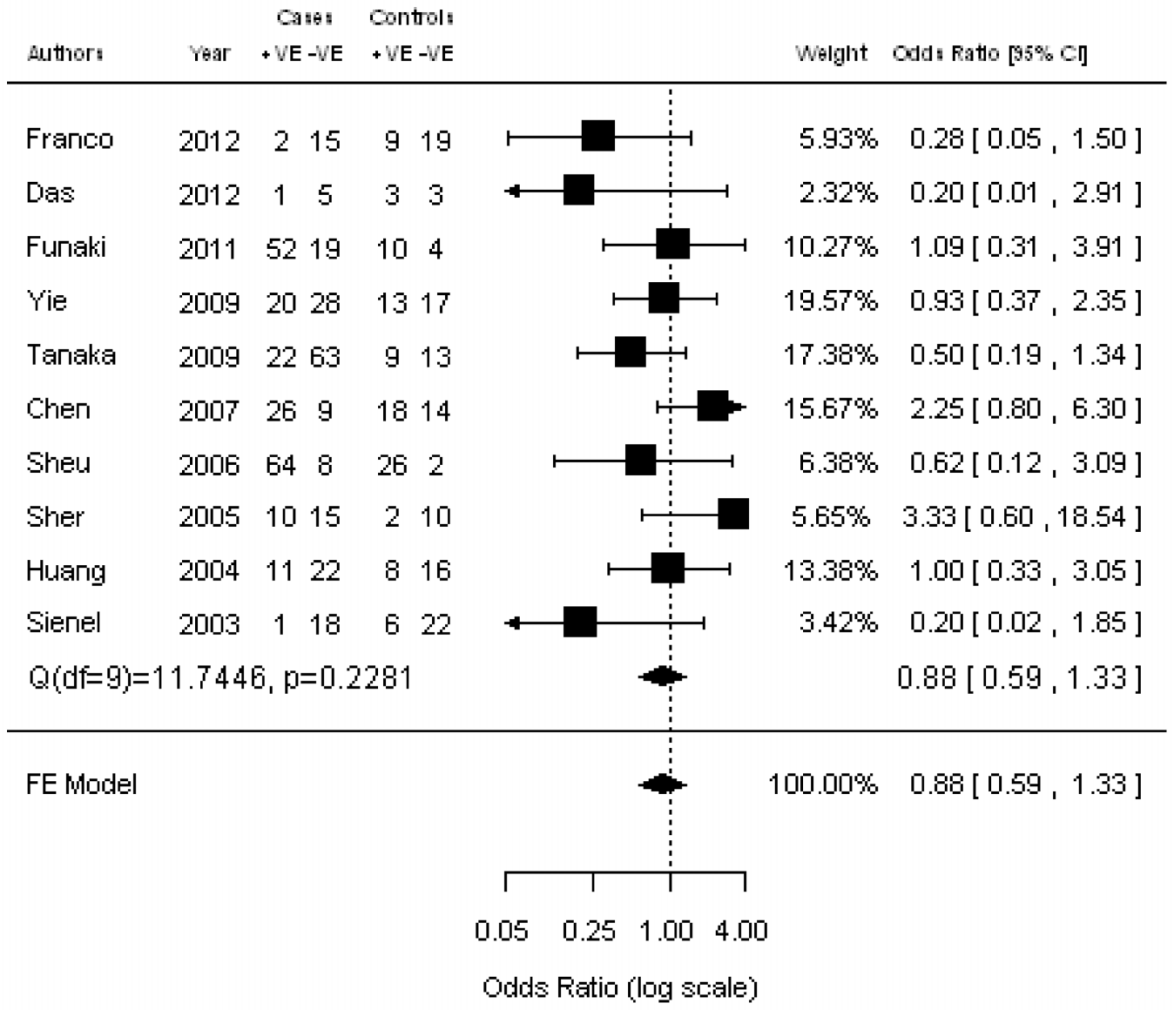
Forrest plot of odds ratios (ORs) was evaluated for association between the presence of CTCs and histology.

**Figure 3:**
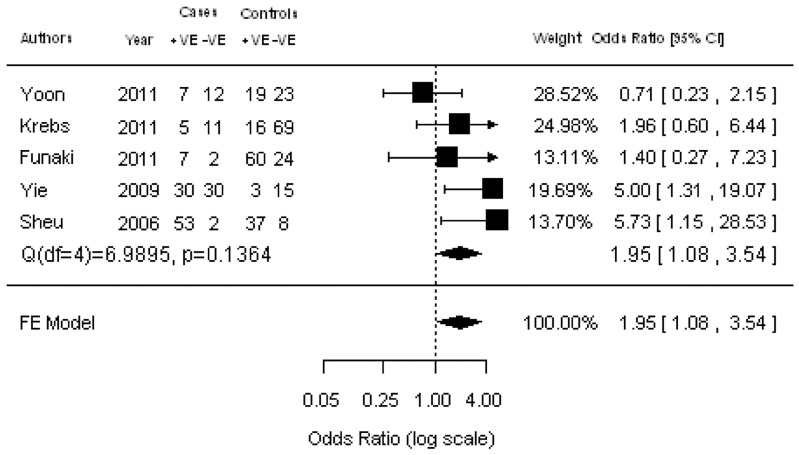
Forrest plot of odds ratios (ORs) was evaluated for association between the presence of CTCs and lymph node metastasis.

**Figure 4:**
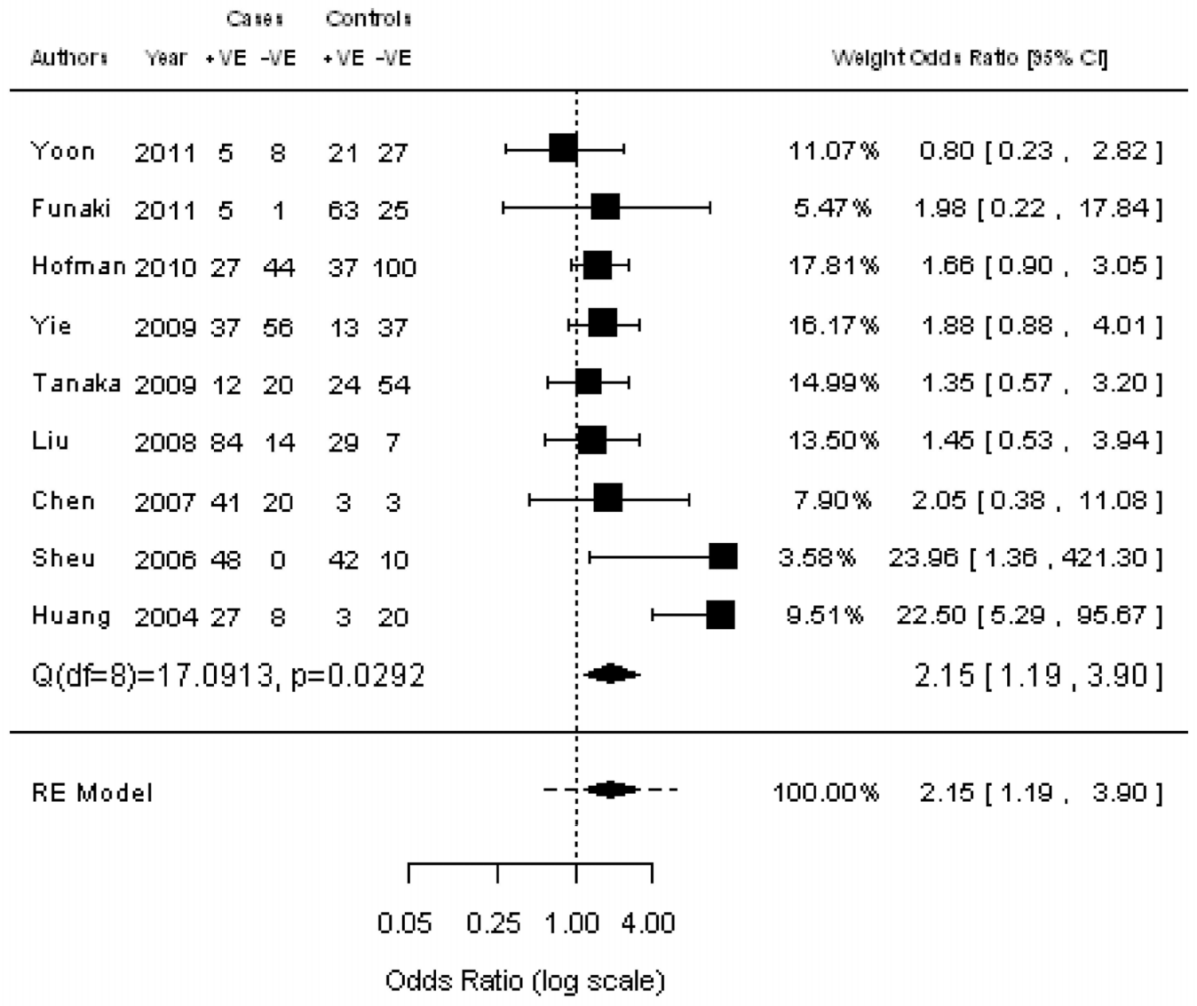
Forrest plot of odds ratios (ORs) was evaluated for association between the presence of CTCs and tumor stage.

Ten studies were available for investigating the relationship between CTC status and histology (adenocarcinoma vs squamous cell carcinoma) (Figure 2). The estimated pooled OR was 0.88 (95% CI: 0.59–1.33; Z = –0.61; *P* = 0.545 fixed-effect), demonstrating that the presence of CTCs was not associated with histology. The heterogeneity and publication bias among studies was not significantly different (Table S1). Five studies assessed the relationship between CTC status and lymph node metastasis (Figure 3). We found that the presence of CTCs was associated with a significantly increased risk of lymph node metastasis in NSCLC patients (pooled OR  = 1.95; 95% CI: 1.08–3.54; Z = 2.20; *P* = 0.027 fixed-effect). There was also a significant association between CTC status and tumor stage (pooled OR  = 2.15; 95% CI: 1.19–3.74; Z = 2.53; *P* = 0.011 random-effect) (Figure 4), showing that the presence of CTCs was associated with a significantly increased risk of progression. Moreover, no significant heterogeneity or publication bias was indicated by Egger’s test.

### Effect of the presence of CTCs on OS in NSCLC

**Figure 5:**
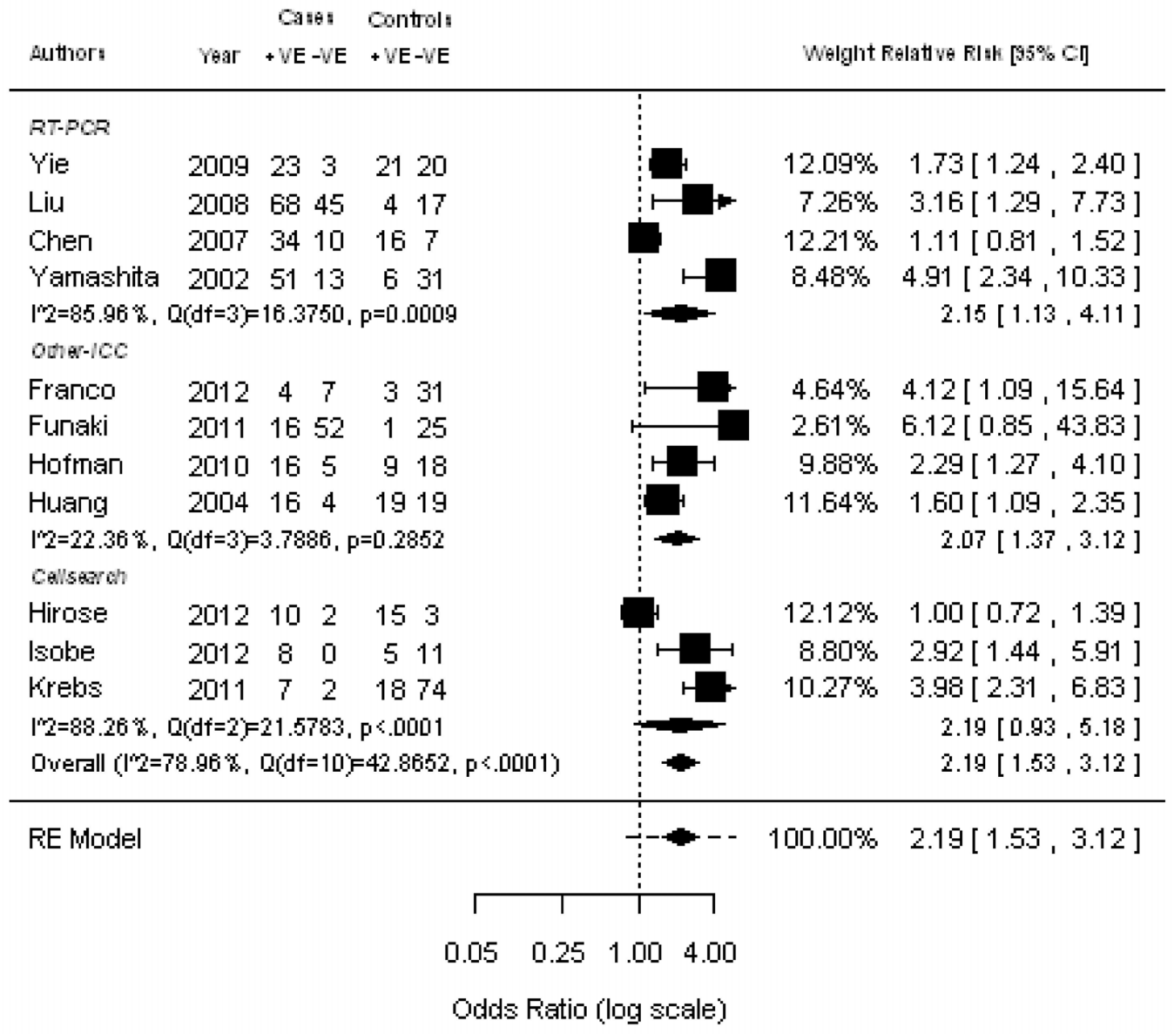
Meta-analysis of relative risk (RR) for the association of the presence of CTCs with overall survival (OS). It showed that patients with CTCs have a poorer survival compared to those without CTCs.

Survival analysis according to CTC status was performed in 13 of 20 studies (65%), accounting for 1072 patients. OS was analyzed in 11 (936 patients) studies. Because the heterogeneity across the studies was less than 0.05 (Q = 54, *P*<0.0001), the estimated pooled RR for studies was calculated using a random-effect model. The pooled RR showed that the presence of CTCs was highly correlated with poor OS (RR  = 2.19; 95% CI: 1.53–3.12; Z = 4.32; *P*<0.0001) (Figure 5). Moreover, using varies detecting techniques sub-group analyses showed similar results. Meta-regression was performed to explore potential sources of heterogeneity using the following covariates: publication year, sample size, tumor stage, and detection method. Univariate analysis did not identify any covariate significantly associated with RR estimates for OS (*P*<0.05) (Table S2). No significant publication bias was detected by Egger’s test.

**Figure 6:**
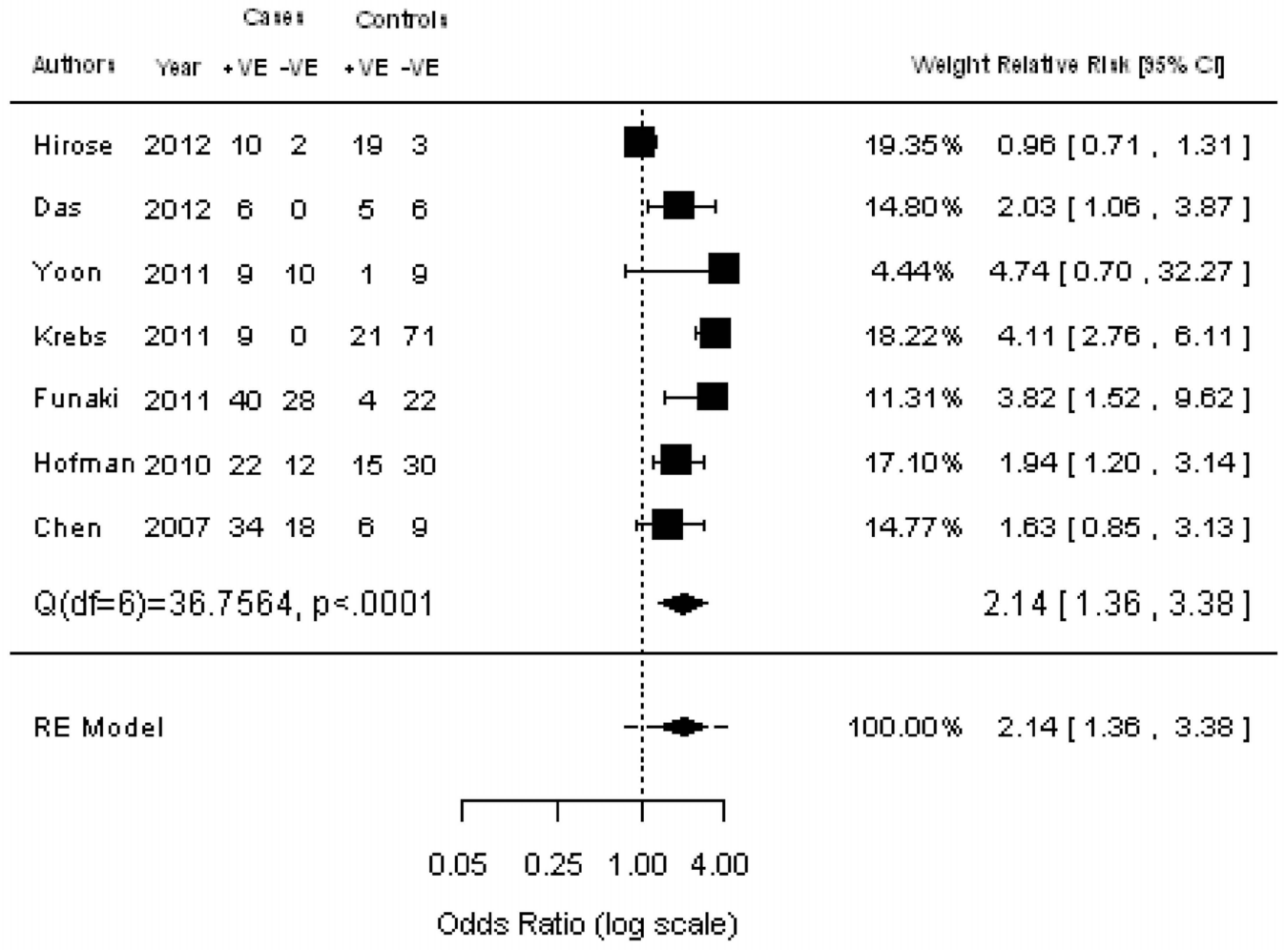
Meta-analysis of RR for the association of the presence of CTCs with progression-free survival (PFS) /disease-free survival (DFS).

With regard to PFS/DFS, seven studies were available that comprised 634 patients. The pooled RR showed that the presence of CTCs was associated with a significantly increased risk of mortality (RR  = 2.14; 95% CI: 1.36–3.38; Z = 3.28; *P*<0.0001 random-effect) (Figure 6). Egger’s test did not identify significant heterogeneity or publication bias.

## Discussion

In this meta-analysis, we provided evidence of an association between the presence of CTCs detected in the blood and clinical outcomes in NSCLC patients. Previous several small-scale studies showed that clinical outcomes showed a poorer survival in patients when CTCs were detected. Therefore, a quantitative meta-analysis of the study outcomes was required. The results of our collective evaluation of the literature on NSCLC indicate that the presence of CTCs could be a prognostic marker.

NSCLC, as opposed to SCLC, is a heterogeneous family with respect to histology and biological characteristics [31]. Different patients with NSCLC and different cells within a tumor express different amounts of marker gene transcripts. In the current study, we showed that the use of different approaches, such as RT-PCR, ICC, and CellSearch, confirmed that CTCs represent a significant meta-risk for both OS and PFS in NSCLC, even after adjustment for publication bias. This is consistent with prior reports of meta-analysis in breast cancer, suggesting that this marker can be developed for clinical applications.

Regarding the number detected, previous studies reported that 30% of NSCLC was ≥1 CTCs per 7.5 ml blood detected by CellSearch, that 15% was ≥5 CTCs, and the CTC count increased significantly with distant metastasis than in patients without [32], [33]. Krebs et al. showed that the number and change in the number of CTCs is a prognostic factor in patients with stage ≥IIIA [25]. Among patients with NSCLC, those with ≥5 CTCs were significantly worse prognosis compared with patients with <5 CTCs. However, few trials had evaluated by subclasstion of enumerating CTCs in patients with NSCLC. Further studies are needed to assess prognostic relevant CTC cut-off levels.

There are limitations to this meta-analysis. First, some data used unadjusted estimates, because not all published papers presented adjusted estimates, resulting in less reliable analysis than direct analysis of variance. However, the conclusions were unlikely to be significantly changed if those studies were adjusted. Second, CTC detection assays varied in our study. In particular, different end points, measurements, and experimental design, may have partly influenced the significance of clinicopathological outcome in survival analyses. Ideally, measurements should be conducted through large prospective studies based on homogeneous published statistics.

In conclusion, available evidence supports that CTCs are associated with tumor stage and lymph node metastasis, but not with histology. Moreover, the presence of CTCs is associated with a poorer outcome than a lack of CTCs, and CTCs are strongly associated with reduced survival. Therapy decisions might be based on CTC results, which could be useful for determining which patients would potentially benefit from adjuvant therapy. These require validation in larger prospectively clinical cohorts.

## Supporting Information

Table S1 **Egger's test of funnel plot asymmetry.**
(DOC)

Table S2 **Results of meta-regression analysis exploring source of heterogeneity with overall survival.**
(DOC)

Checklist S1 **PRISMA checklist.**
(DOC)
