# Correction: Prognostic Significance of Circulating Tumor Cells in Non-Small-Cell Lung Cancer Patients: A Meta-Analysis

**DOI:** 10.1371/annotation/6633ed7f-a10c-4f6d-9d1d-9c1245822eb7

**Published:** 2014-01-03

**Authors:** Jianwei Wang, Ke Wang, Jianjun Xu, Jian Huang, Tao Zhang

The name of the first author is incorrect. The correct name is: Jianwei Wang. The correct Citation is: Wang J, Wang K, Xu J, Huang J, Zhang T (2013) Prognostic Significance of Circulating Tumor Cells in Non-Small-Cell Lung Cancer Patients: A Meta-Analysis. PLoS ONE 8(11): e78070.

doi:10.1371/journal.pone.0078070. The correct abbreviation in the Author Contributions Statement is: JW. 

