# Supplementary material for: Prognostic Significance of Circulating Tumor Cells in Non-Small-Cell Lung Cancer Patients: A Meta-Analysis
Source: PLoS One. 2013 Nov 4;8(11):e78070. doi: 10.1371/journal.pone.0078070 (PMC3817175; doi:10.1371/journal.pone.0078070)
Supplement: Table S1 — Egger's test of funnel plot asymmetry. (DOC) [file pone.0078070.s001.doc]

**Table S1 Egger's test of funnel plot asymmetry.**

| Clinicopathological parameters | t value | df | *P* value |
| --- | --- | --- | --- |
| Histology | -0.72 | 7 | 0.49 |
| Lymph node metastasis | 1.17 | 3 | 0.32 |
| Stage | 1.36 | 7 | 0.21 |
| Overall survival | 0.19 | 9 | 0.85 |
| PFS | 0.79 | 4 | 0.47 |
