# Supplementary material for: Prognostic Significance of Circulating Tumor Cells in Non-Small-Cell Lung Cancer Patients: A Meta-Analysis
Source: PLoS One. 2013 Nov 4;8(11):e78070. doi: 10.1371/journal.pone.0078070 (PMC3817175; doi:10.1371/journal.pone.0078070)
Supplement: Table S2 — Results of meta-regression analysis exploring source of heterogeneity with overall survival. (DOC) [file pone.0078070.s002.doc]

**Table S2 Results of meta-regression analysis exploring source of heterogeneity with overall survival.**

| Covariates | Univariate analysis | | |
| --- | --- | --- | --- |
|  | Coefficient | SE | *P* value |
| Publication year | 0.05 | 0.16 | 0.75 |
| Sample size | 0.09 | 0.24 | 0.69 |
| Tumor stage | -0.27 | 0.91 | 0.77 |
| Detection method | -0.20 | 0.61 | 0.74 |

Note: The dependent variable is the RR for overall survival (OS) from each study. Weights have been assigned according to the estimated variance of RR. SE, standard error of the coefficient.
